# Atypical hemolytic uremic syndrome

**DOI:** 10.1186/1750-1172-6-60

**Published:** 2011-09-08

**Authors:** Chantal Loirat, Véronique Frémeaux-Bacchi

**Affiliations:** 1 Assistance Publique-Hôpitaux de Paris, Hôpital Robert Debré; Université Paris VII; Pediatric Nephrology Department; Paris, France; 2 Assistance Publique-Hôpitaux de Paris, Hôpital Européen Georges Pompidou; Biological Immunology Department; Paris, France

**Keywords:** Atypical hemolytic uremic syndrome, C3, factor H, factor I, factor B, membrane cofactor protein, thrombomodulin, plasma infusion, plasma exchange, eculizumab, kidney transplantation, combined liver-kidney transplantation

## Abstract

Hemolytic uremic syndrome (HUS) is defined by the triad of mechanical hemolytic anemia, thrombocytopenia and renal impairment. Atypical HUS (aHUS) defines non Shiga-toxin-HUS and even if some authors include secondary aHUS due to *Streptococcus pneumoniae *or other causes, aHUS designates a primary disease due to a disorder in complement alternative pathway regulation. Atypical HUS represents 5 -10% of HUS in children, but the majority of HUS in adults. The incidence of complement-aHUS is not known precisely. However, more than 1000 aHUS patients investigated for complement abnormalities have been reported. Onset is from the neonatal period to the adult age. Most patients present with hemolytic anemia, thrombocytopenia and renal failure and 20% have extra renal manifestations. Two to 10% die and one third progress to end-stage renal failure at first episode. Half of patients have relapses. Mutations in the genes encoding complement regulatory proteins factor H, membrane cofactor protein (MCP), factor I or thrombomodulin have been demonstrated in 20-30%, 5-15%, 4-10% and 3-5% of patients respectively, and mutations in the genes of C3 convertase proteins, C3 and factor B, in 2-10% and 1-4%. In addition, 6-10% of patients have anti-factor H antibodies. Diagnosis of aHUS relies on 1) No associated disease 2) No criteria for Shigatoxin-HUS (stool culture and PCR for Shiga-toxins; serology for anti-lipopolysaccharides antibodies) 3) No criteria for thrombotic thrombocytopenic purpura (serum ADAMTS 13 activity > 10%). Investigation of the complement system is required (C3, C4, factor H and factor I plasma concentration, MCP expression on leukocytes and anti-factor H antibodies; genetic screening to identify risk factors). The disease is familial in approximately 20% of pedigrees, with an autosomal recessive or dominant mode of transmission. As penetrance of the disease is 50%, genetic counseling is difficult. Plasmatherapy has been first line treatment until presently, without unquestionable demonstration of efficiency. There is a high risk of post-transplant recurrence, except in MCP-HUS. Case reports and two phase II trials show an impressive efficacy of the complement C5 blocker eculizumab, suggesting it will be the next standard of care. Except for patients treated by intensive plasmatherapy or eculizumab, the worst prognosis is in factor H-HUS, as mortality can reach 20% and 50% of survivors do not recover renal function. Half of factor I-HUS progress to end-stage renal failure. Conversely, most patients with MCP-HUS have preserved renal function. Anti-factor H antibodies-HUS has favourable outcome if treated early.

## Disease name and synonyms

A classification of hemolytic uremic syndrome (HUS) and thrombotic thrombocytopenic purpura (TTP)--the two main variants of thrombotic microangiopathies (TMA)-and related disorders according to etiology has been proposed by the European Pediatric Research Group for HUS [1]. In common medical language, the names typical or post-diarrheal (D+) HUS describe the most frequent form of HUS in children, due to Shiga-toxin (Stx) producing *Escherichia coli *(STEC), mostly *E coli *0157:H7. By opposition, the name atypical HUS (aHUS) has been historically used to describe any HUS not due to STEC, thus including:

i) "Secondary" aHUS, due to a variety of causes, including infectious agents different from STEC, mostly *Streptococcus pneumoniae *(*S pneumoniae*) (via neuraminidase of *S pneumoniae *and T antigen exposure), human immunodeficiency virus and H1N1 influenza A, malignancy, cancer chemotherapy and ionizing radiation, bone marrow or solid organ transplantation, calcineurin inhibitors, sirolimus or anti vascular endothelial growth factor (VEGF) agents, pregnancy, HELLP (Hemolytic anemia, elevated Liver enzymes, and Low Platelets) syndrome, malignant hypertension, glomerulopathies, systemic diseases (systemic lupus erythematous and antiphospholipid antibody syndrome, sclerodermia) or, in children, methyl malonic aciduria with homocystinuria, cblC type, a rare hereditary defect of cobalamine metabolism [1-14]. Of note, it is now acknowledged that using the aHUS terminology rather than an etiological-based denomination (e.g. *S pneumoniae*-HUS) is inadequate [1].

ii) aHUS classified as "primary", at least until the years 2000, as no exogenous cause was identified and the mechanism was unknown. However, it was recognized nearly four decades ago that this form of HUS could be familial, touching members of the family several years apart [15]. This is why it is also described as hereditary HUS. During the last decade, this form of aHUS has been demonstrated to be a disease of complement dysregulation. Therefore it is now described as "complement dysregulation -associated aHUS" or, for abbreviation, "complement-HUS". Of note, most authors, including ourselves for this review, now use the aHUS denomination to designate only complement-HUS [16].

Another denomination for aHUS has been non-post-diarrheal (D-) HUS, because the prodromal bloody diarrhea characteristic of STEC-HUS was rarely the predominant symptom. However, as gastroenteritis is a frequent trigger of complement-HUS episodes [17,18], this terminology of (D-) HUS should be withdrawn. In practice, some publications on (D-) HUS/aHUS in children include *S pneumoniae *- HUS, a frequent category in children [19]. Also, some publications on complement-HUS include some secondary aHUS [18,20], while others exclude the various causes indicated above (except pregnancy and contraceptive pill) [17,21-23]. This may explain differences in results. Last, as HUS and TTP share in common hemolytic anemia and thrombocytopenia, with predominant central nervous system (CNS) involvement in TTP and predominant renal involvement in HUS, both diseases are often grouped under the denomination TTP/HUS. This is also due to the possible overlap of symptoms, with CNS involvement in HUS and renal involvement in TTP. TTP and aHUS can now be differentiated according to their different physiopathology i.e. deficiency of the von Willebrand cleaving protease, ADAMTS (A Disintegrin And Metalloprotease with ThromboSpondin type 1 repeats) 13, in TTP (commonly acquired via circulating autoantibodies in adults and rarely inherited (Upshaw-Schulman syndrome) via recessive ADAMTS-13 mutations in neonates or young children) and complement dysregulation in aHUS. However, biological investigations may not confirm the clinical diagnosis as at least 10-25% of TTP patients have normal ADAMTS13 activity and 30% of aHUS patients have no complement anomalies, suggesting the presence of unknown physiopathological mechanisms [16,24,25].

## Definition

HUS is defined by the triad of mechanical, non-immune (negative Coombs test, except false positivity in *S pneumoniae*-HUS, see section Differential diagnosis) hemolytic anemia (hemoglobin < 10 g/dL) with fragmented erythrocytes (schizocytes), thrombocytopenia (platelets < 150.000/mm^3^) and renal impairment (serum creatinine > upper limit of normal for age). High lactate deshydrogenase (LDH) and undetectable haptoglobin levels confirm intra vascular hemolysis. The underlying histological lesion is TMA, characterized by thickening of arteriole and capillary walls, with prominent endothelial damage (swelling and detachment), subendothelial accumulation of proteins and cell debris, and fibrin and platelet-rich thrombi obstructing vessel lumina. TMA predominantly affects the renal microvasculature, although the brain, heart, lungs and gastrointestinal tract may be involved. When none of the etiologies indicated in the preceding chapter is present, the diagnosis of primary aHUS, now demonstrated to be a disease of complement dysregulation, is most probable. Our aim is to review the tremendous progress performed during the last decade in the understanding of this disease, and to show how this new knowledge has opened the way to new therapies.

## Epidemiology

The incidence of aHUS is estimated in the USA to 2 per million, a number calculated from the incidence of (D-) HUS in children, including *S pneumoniae -*HUS [19]. In reality, the incidence of complement-aHUS is not known precisely. However, more than 1000 aHUS patients investigated for complement abnormalities have been reported from five European registries or series [17,18,20-22,26-28] and one from the USA [23].

## Clinical Description

### Gender and age at onset

aHUS is equally frequent in boys and girls when onset occurs during childhood [17], while there is a female preponderance in adults [20]. aHUS occurs at any age, from the neonatal period to the adult age (extremes: 1 day to 83 years [17,18]. Onset during childhood (≤ 18 years) appears slightly more frequent than during adulthood (approximately 60% and 40% respectively) [18,21]. Seventy per cent of children have the first episode of the disease before the age of 2 years and approximately 25% before the age of 6 months [17]. Therefore, onset before the age of 6 months is strongly suggestive of aHUS, as less than 5% of STEC-HUS occur in children less than 6 months [29,30] and personnal communication of Lisa King, Institut de Veille Sanitaire, St Maurice, France, with permission].

### Triggering events

An infectious event, mainly upper respiratory tract infection or diarrhea/gastroenteritis, triggers onset of aHUS in at least half of patients [18], up to 80% in pediatric cohorts [17,31]. Interestingly, diarrhea preceded aHUS in 23% and 28% of patients in the French pediatric [17] and the Italian adult and pediatric [18] cohorts respectively, showing that the classification of HUS as (D+) or (D-) may be misleading and that post-diarrheal onset does not eliminate the diagnosis of aHUS. Other triggers such as varicella [32], H1N1 influenza [6,33-35] and, interestingly, STEC-diarrhea [17,18,36,37] have been reported in patients who were investigated for aHUS because of a fulminant course, a familial incidence of the disease or the subsequent occurrence of relapses. Pregnancy is a frequent triggering event in women [18,38,39]: 20% of women with aHUS experience the disease, mostly the inaugural episode, at pregnancy, 80% of them during the post-partum period [39]. These observations highlight the difficulty to define the limit between aHUS triggered by an incidental event and secondary HUS.

### Presenting features

Onset is generally sudden. Symptoms in young children are pallor, general distress, poor feeding, vomiting, fatigue, drowsiness and sometimes oedema. Adults complain of fatigue and general distress. Most patients have the complete triad of HUS at first biological investigation: hemoglobin < 10 g/dL (not exceptionally as low as 3-4 g/dL), platelets count < 150 000/mm^3 ^(generally between 30 000 and 60 000/mm^3^, with no or little risk of bleeding complications), and renal insufficiency (serum creatinine > normal value for age), with or without anuria or reduced urine volume, proteinuria if diuresis is maintained. The presence of schizocytes, undetectable haptoglobin and high LDH levels confirm the microangiopathic intravascular origin of hemolysis. If diagnosis is delayed, life-threatening hyperkaliemia (≥ 6 mmol/L), acidosis (serum bicarbonates < 15 mmol/L) and volume overload with arterial hypertension and hyponatremia (< 125 mmol/L) may be observed. Arterial hypertension is frequent and often severe, due both to volume overload in case of oliguria/anuria and to hyperreninemia secondary to renal TMA. Cardiac failure or neurological complications (seizures) due to hypertension are possible. Half of children and the majority of adults need dialysis at admission.

Extra renal manifestations are observed in 20% of patients [17,18]. The most frequent is CNS involvement (10% of patients) manifested by irritability, drowsiness, seizures, diplopia, cortical blindness, hemiparesis or hemiplegia, stupor, coma. Brain magnetic resonance imaging (MRI) is useful to differenciate CNS complications due to arterial hypertension (reversible posterior leukoencephalopathy syndrome with posterior white matter hyper intensity predominant in the parieto-occipital regions) and those due to cerebral TMA (on FLAIR and T2 sequences, bilateral and symetrical hyperintensities of the basal ganglia, cerebral pedunculas, caudate nuclei, putamens, thalami, hippocampi, insulae and possibly brainstem [40]. Myocardial infarction due to cardiac microangiopathy has been reported in approximately 3% of patients and explains cases of sudden death [18,41]. Distal ischemic gangrene leading to amputation of fingers and toes can also occur [42]. Approximately 5% of patients present with a life-threatening multivisceral failure due to diffuse TMA, with CNS manifestations, cardiac ischemic events, pulmonary hemorrhage and failure, pancreatitis, hepatic cytolysis, intestinal bleeding [17,18].

Some patients (approximately 20% of children [17] and a similar percentage in adults) have a progressive onset with subclinical anemia and fluctuating thrombocytopenia during weeks or months and preserved renal function at diagnosis. They may go to remission and subsequently have an acute relapse, or they develop progressive hypertension, proteinuria that may induce nephrotic syndrome, and increase of serum creatinine over several weeks or months. Some patients have no anemia or thrombocytopenia and the only manifestations of renal TMA are arterial hypertension, proteinuria and a progressive increase of serum creatinine.

In children, age, clinical context and symptoms at presentation most often allow to differenciate patients as having TTP or HUS, and, if HUS most likely, as having post-diarrheal STEC-HUS, invasive *S pneumoniae *infection or complement-HUS. On the opposite, clinical presentation is more confusing in adults and complement-HUS has to be suspected whatever the clinical context.

## Pathogenesis

As early as 1970-1980, it had been noticed that some patients with aHUS had low C3 plasma levels [43]. Impressive progress has been done during the last decade, showing that 4 regulatory proteins of the complement alternative pathway, complement factor H (CFH), membrane cofactor protein (MCP or CD46), factor I (CFI) and thrombomodulin (THBD) and 2 proteins of the C3 convertase, C3 and factor B (CFB), had a role in the pathogenesis of aHUS.

### Complement and its regulation

**Figure 1:**
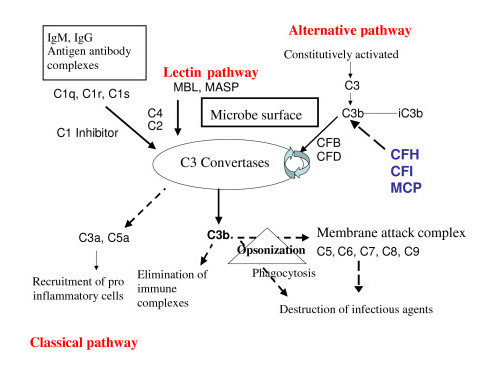
**The 3 pathways of complement activation**. Classical, lectin and alternative pathways converge at the point of C3 activation. The lytic pathway then leads to the assembly of the membrane attack complex which destroys infectious agents. Regulators of the alternative pathway CFH, CFI and MCP cooperate to inactivate endothelial cell surface-bound C3b, thus protecting endothelial cells from complement attack. CFH: factor H; CFI: factor I; CFB: factor B; CFD: factor D; MCP: membrane cofactor protein.

**Figure 2:**
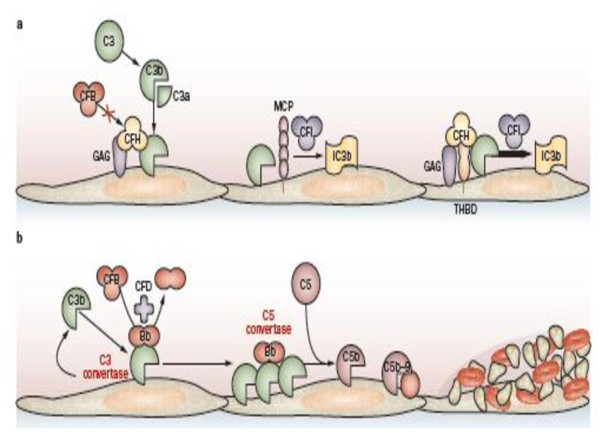
**Regulated and deregulated activation of the alternative complement pathway**. Figure and comments reproduced from Zuber *et al *[131]. **a) **CFH competes with CFB to bind C3b, which hampers the generation of C3 convertase. CFH binds to glycosaminoglycans on the endothelial surface and factors, such as MCP, can act as a cofactor for the CFI-mediated cleavage of C3b to generate iC3b (inactivated C3b). THBD binds to C3b and CFH and might accelerate the CFI-mediated inactivation of C3b. **b**) Uncontrolled activation of the alternative complement pathway leads to the generation of the membrane-attack complex (C5b-9) through the actions of CFB, CFD and through the generation of C3 convertase and C5 convertase. The resulting injury and activation of endothelial cells initiates a microangiopathic thrombotic process. CFH: factor H; CFI: factor I; CFB: factor B; CFD: factor D; MCP: membrane cofactor protein; THBD: thrombomodulin.

Complement is the main system for defense against bacteria. It is activated by three pathways: the classical pathway, the lectin pathway and the alternative pathway [44] (Figure 1). These three pathways converge at the point of cleavage of C3. While the activation of the classical and the lectin pathways occurs after binding to immune complexes or microorganisms respectively, the alternative pathway is continually activated and generates C3b which binds indiscriminately to pathogens and host cells. On a foreign surface, such as a bacterium, C3b binds CFB, which is then cleaved by Factor D to form the C3 convertase C3bBb. The C3bBb produces exponential cleavage of C3 (amplification loop) and the formation of the C5 convertase (C3bBb(C3b)n). C5b component, generated by C5 cleavage, participates in the assembly of the membrane-attack complex (MAC) C5b9, which induces opsonization, phagocytosis and lysis of bacteria (Figure 1). This reaction is normally strictly controlled at the host cell surfaces, which are protected from the local amplification of C3b deposits by several complement regulatory proteins: CFH (a plasma glycoprotein, cofactor for CFI), CFI (a plasma serine protease which cleaves and inactivates C3b to form iC3b in the presence of cofactors, including MCP (a non-circulating glycoprotein anchored in all cell membranes except red blood cells), and possibly THBD, an endothelial glycoprotein with anticoagulant, anti-inflammatory, and cytoprotective properties but also a regulator of the complement system [45]. In the presence of CFH, the competition between CFH and CFB binding to C3b also limits the formation of the C3 convertase. When CFH is bound to the C3b attached on cell surface, CFB can no longer form the C3 convertase (Figure 2).

**Figure 3:**
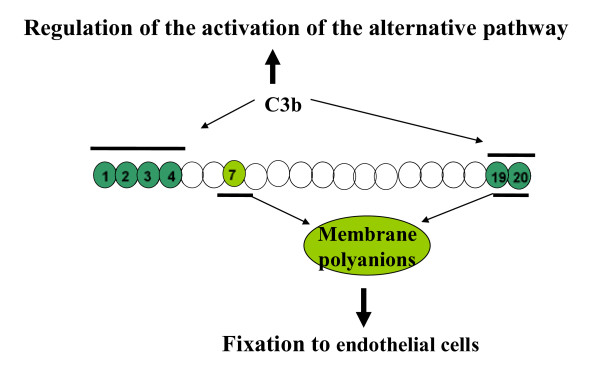
**Factor H**. Factor H is constituted by 20 short consensus repeats (SCR). The two binding sites for C3b are in SCR 1-4 and 19-20. The binding sites for polyanions of cell surface (vascular endothelium) are in SCR 7 and 19-20. SCR 1-4 are involved in the binding of CFH to circulating C3b i.e. the regulation of complement alternative pathway activation in the fluid phase. SCR 7 and 19-20 are involved in the binding of CFH to polyanionic surface-bound C3b i.e. the regulation of complement alternative pathway activation at the endothelial cell surface.

CFH is the most important protein for the regulation of the alternative pathway. CFH consists of 20 short consensus repeats (SCRs) (Figure 3) and contains at least two C3b-binding sites. The first binding site to C3b, which regulates fluid phase alternative pathway amplification, is located within the N-terminal SCR1-4. The second C3b-binding site is located in SCR19-20, in the C-terminal domain. CFH also contains two polyanion-binding sites in SCR7 and SCR19-20. Endothelial cells are rich in polyanionic molecules e.g. glycosaminoglycans. The protection of the host cells depends on the inactivation of surface-bound C3b secondary to the binding of the CFH to the surface-bound C3b. All recent studies clearly demonstrate the role of SCR19-20 in the protection of endothelial cells [46-48]. The four proteins CFH, CFI, MCP and THBD cooperate locally to cleave C3b to an inactive molecule (iC3b). It has been proposed that the mutations identified in aHUS patients in the genes *CFH*, *MCP, CFI *and *THBD *induce a defect of the protection of endothelial cells towards complement activation [46,49-51]. Altogether, all identified genetic defects end up in an amplified generation of C3 convertase and secondarily the generation of C5 convertase and thus the cleavage of C5. This results in increased liberation of C5a and MAC at the endothelial cell surface, causing additional endothelial cell damage with exposure of the subendothelial matrix and thrombus formation. This produces platelet consumption and red cell damage (Figure 2). Any alteration of the endothelial cells (inflammation, apoptosis) may participate actively to this mechanism. In addition, a prominent role of CFH in modulating platelet structure and function has been demonstrated [52,53]. C-terminal CFH mutants have a reduced ability to bind to platelets, resulting in complement activation on the surface of platelets. This in turn causes platelet activation and aggregation and release of tissue-factor expressing microparticules and participates to the formation of thrombi within the microcirculation [52].

This physiopathological model is corroborated by transgenic animal models. Mice which express CFH variant lacking the C-terminal 16-20 domain develop HUS similar to the human disease, including TMA glomerular lesions [54]. In this mouse model, CFH regulates C3 activation in the plasma, but fails to bind to endothelial cells, similar to mutant CFH of aHUS patients. Interestingly, this mouse model permitted to demonstrate the key role of complement C5 in the development of HUS. When these mice were crossed with mice deficient in C5, a complete protection from glomerular injury and HUS was observed [55]. This demonstrates that activation of C5, probably by unregulated production of C5 convertase, is essential for the development of aHUS.

### Complement dysregulation in aHUS

#### CFH mutations

**Figure 4:**
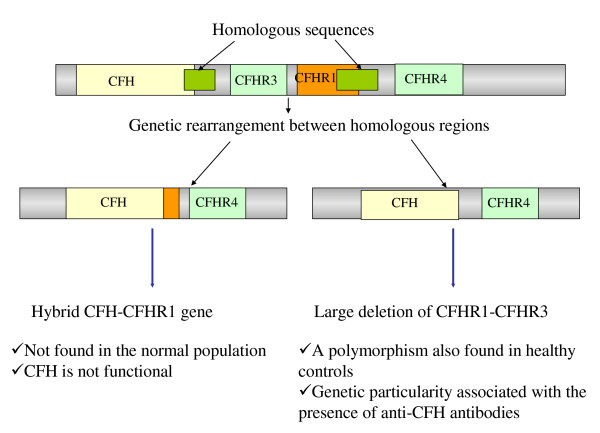
***Complement factor H-related *(*CFHR*) genes and their abnormalities in atypical hemolytic uremic syndrome: genetic rearrangements between *CFH *and contiguous genes *CFHR1 *and *CFHR3 *or deletion of *CFHR1*-*R3***.

They were the first identified. A decrease of plasma C3 level was first reported in 1973 in 5 patients with severe HUS [43]. The association of aHUS with a low CFH plasma level was then reported for the first time in 1981 [56]. However, it is only in 1998 that Warwicker et al, by genetic study of 3 families, could establish the link between aHUS and the RCA (regulators of complement activation) locus in chromosome 1q32, where the genes of CFH and MCP are located. The first candidate gene studied was CFH, and a heterozygous mutation in SCR20 was first demonstrated [57]. Subsequently, several groups showed that a number of patients with aHUS had, despite normal plasma levels of CFH, mutations in CFH gene, mainly in SCR19 and 20 [18,21,31,46]. Presently, more than 100 different mutations of CFH have been identified in adults and children with sporadic or familial HUS [58]. More than 50% of CFH mutations are in SCR20 [38]. Functional studies to analyse the interaction between CFH and its ligands (C3b, glycosaminoglycans, heparin and endothelial cells) frequently demonstrate alteration of the binding of CFH19-20 mutants [50,59-61]. Some mutations (named type 1 mutations) are associated with a quantitative deficiency in CFH (decreased CFH plasma levels), but many, including the majority of mutations in SCR19 and 20, are associated with normal plasma levels of CFH, the mutant CFH being functionally deficient (type 2 mutations). Last, *CFH *is in close proximity to the genes *CFHR1-5 *encoding five CFH-related proteins (Figure 4). *CFH *and *CFH-Rs *share a high degree of sequence identity, which predisposes to complex rearrangements leading to non-functional CFH, such as hybrid CFH which has lost SCR19 and 20 due to the combination of the first 21 N-terminal exons of *CFH *(encoding SCR1 to 18) and the 2 C-terminal exons of *CFH-R1 *[62,63] (Figure 4). Homozygous mutations can be observed. These patients have very low C3 and CFH plasma concentrations. But most mutations are heterozygous. Plasma C3 level is decreased in 30% to 50% of patients with heterozygous mutant CFH, and more frequently in type 1 than in type 2 mutations. C3 plasma level may be decreased while CFH level is normal and vice versa [18,31,64]. (Table 1 and Table 2). Mutations in *CFH *are the most frequent genetic abnormality in aHUS patients as they account for 20 to 30% of cases (Table 3) [18,23,31,50]. The frequency of hybrid CFH is approximately 1-3% in aHUS patients screened with *CFH *Multiplex Ligation dependent Probe Amplification (MLPA) (see section Diagnostic methods) [18].

**Table 1:** Percentage of patients with decreased C3 plasma concentration in the various subgroups of atypical hemolytic uremic syndrome

	*CFH *mutation	*CFI *mutation	*MCP *mutation	*C3 *mutation	*CFB *mutation	*THBD *mutation	Anti -CFH Ab	None
**Decreased C3 concentration** **(< 2SD)** **(% patients)**	30-50%	20-30%	0-27%	70-80%	100%	50%	40-60%	up to 20%

Normal C3 plasma concentration does not eliminate the presence of a mutation in the complement system or of anti- CFH antibodies. Conversely, decreased C3 level signs the presence of a complement abnormality.

CFH: factor H; CFI: factor I; MCP: membrane cofactor protein; CFB: factor B; THBD: thrombomodulin; Ab, antibodies.

**Table 2:** Plasma concentration of C3, C4, CFH, CFI and CFB and expression of MCP in the various subgroups of atypical hemolytic uremic syndrome

	Protein level or expression					
	
	C4	C3	CFH	CFI	CFB	MCP
***CFH *mutation**	N	Normal (decreased)	Normal (decreased)	Normal	Normal (decreased)	Normal

***CFI *mutation**	N	Normal (decreased)	Normal	Normal (decreased)	Normal (decreased)	Normal

***MCP *mutation**	N	Normal (decreased)	Normal	Normal	Normal	Decreased (normal)

***CFB *mutation**	N	Decreased	Normal	Normal	Normal (decreased)	Normal

***C3 *mutation**	N	Decreased	Normal	Normal	Normal (decreased)	Normal

***THBD *mutation**	N	Normal or decreased	ND	ND	ND	Normal

**Anti-CFH Ab**	N	Decreased (normal)	Normal (decreased)	Normal	Normal (decreased)	Normal

Very low C3 levels are observed in patients with homozygous *CFH *mutation (complete CFH deficiency) or compound heterozygous *CFH *mutation, and in patients with *CFB *or *C3 *gain-of-function mutations. In most of the other patients, C3 concentration is mildly decreased or normal. Undetectable CFH concentrations are observed only in patients with homozygous *CFH *mutation. Decreased CFH concentration can be observed in patients with heterozygous type 1 *CFH *mutation, and during flares of anti-CFH antibodies-HUS. Decreased C4 plasma levels have been reported in zero to a few % of patients of the various subgroups [18].

"Normal" and "decreased" without brackets means most frequently normal or decreased, within brackets means possible but not frequent. "Normal or decreased" without brackets means normal or decreased is equally frequent. CFH: factor H; CFI: factor I; MCP: membrane cofactor protein; CFB: factor B; THBD: thrombomodulin; Ab, antibodies. ND: not documented.

**Table 3:** Main clinical characteristics of patients with atypical hemolytic uremic syndrome according to complement abnormality

Gene or subgroup	Frequency in aHUS	Minimal age at onset		Risk of death or ESRD at 1^st ^episode or within < 1 y	Risk of relapses	Risk of recurrence after renal transplantation	Plasma therapy indicated
		Children	Adults				
***CFH***	20-30%	Birth	any age	50-70%	50%	75-90%	Yes

***CFI***	4 -10%	Birth	any age	50%	10-30%	45-80%	Yes

***MCP***	5 -15%	> 1 y	any age	0-6%	70-90%	< 20%	Questionable

***C3***	2 -10%	7 m	any age	60%	50%	40-70%	Yes

***CFB***	1-4%	1 m	any age	50%	3/3 not in ESRD	100%	Yes

***THBD***	3 -5%	6 m	rare	50%	30%	1 patient	Yes

**Anti-CFH Ab**	6%	Mostly 7-11 y		30-40%	40-60%	Yes if high Ab titer	Yes (+ IS)

CFH: factor H; CFI: factor I; MCP: membrane cofactor protein; CFB: factor B; THBD: thrombomodulin; Ab, antibodies; ESRD: end stage renal disease; IS: immunosuppressive treatment.

#### Anti-CFH autoantibodies

An acquired dysfunction of CFH due to anti-CFH antibodies was first described in 2005 [65]. The anti-CFH IgG bind to CFH SCR19 and 20 and thus inhibit CFH binding to C3b and cell surfaces [66-68]. Ninety per cent of patients with anti CFH-antibodies have a complete deficiency of CFHR1 and CFHR3 associated to a homozygous deletion of *CFHR1 *and *CFHR3 *(Figure 4), suggesting that this deletion has a pathogenic role in the development of anti-CFH autoantibodies [[27,37,69-71]. Patients with anti-CFH antibodies can also have mutations [18,71]: out of 13 patients with anti-CFH antibodies, 5 had mutations in *CFH *, *CFI*, *MCP *or *C3 *[71]. Plasma C3 concentration is decreased in 40 to 60% of patients with anti-CFH antibodies [18,37], and is lower in patients with high titers of anti-CFH IgG than in those with moderate titers [37]. CFH plasma concentration was decreased at disease onset in 22% of patients studied by Dragon-Durey *et al*, not correlated with anti-CFH IgG titers [37] (Table 1 and Table 2). Overall, anti-CFH antibodies account for approximately 6% of aHUS, mainly in children (10-12% of aHUS in children) (Table 3) [18,37,71,72].

#### MCP mutations

Richards et al in 2003 were the first to report mutations of *MCP *in seven aHUS patients from 3 families [73]. More than 40 different mutations in *MCP *have now been identified in patients with aHUS [38,50,58,74]. The mutant MCP has low C3b-binding and cofactor activity [21,75]. Most of the mutations are heterozygous, some are homozygous or compound heterozygous. Most patients present a decreased expression of MCP on peripheral leucocytes (granulocytes or mononuclear cells), an important diagnostic test. Less frequently, the expression of MCP is normal, but the protein is dysfunctional. Of note, we observed that MCP expression can be decreased transiently at the acute phase of any type of HUS, therefore this is not strictly synonymous of MCP-HUS, unless the decrease persists after resolution of the acute phase (unpublished data from V.Frémeaux-Bacchi). C3 levels in MCP-mutated patients are most often normal, a logic issue as MCP mutations are not expected to activate complement in the fluid phase. However, decreased C3 concentrations have been reported in up to 27% of patients from the Italian Registry [18] (Table 1 and Table 2). It is likely that some of the MCP-mutated patients with decreased C3 have another mutation responsible of the activation of complement in the fluid phase. *MCP *mutations are more frequent in children than in adults [18] and account for 5-15% of aHUS patients (Table 3) [17,18,20,23].

#### CFI mutations

Mutations in *CFI *were first described in 2004 in 3 patients with aHUS [76]. Approximately 40 mutations in *CFI *have been reported in patients with aHUS, all heterozygous [28,58,77-79]. CFI mutations either induce a default of secretion of the protein or disrupt its cofactor activity, with altered degradation of C3b/C4b in the fluid phase and on surfaces [28,78,79]. Plasma C3 concentration is decreased in 20-30% of patients and CFI concentration in approximately one third of patients. C3 level can be decreased while CFI level is normal or vice versa [18,28,31,76] (Table 1 and Table 2). The frequency of *CFI *mutations in aHUS patients varies from 4% to 10% according to series. Thirty per cent of patients with *CFI *mutations carry at least one additional known genetic risk factor for aHUS [28].

#### CFB mutations

In 2007 and 2009, Goicoechea de Jorge *et al *[80] and Roumenina *et al *[81] reported four heterozygous mutations in *CFB *in aHUS-patients. These mutations are gain of function mutations, ending-up in a "super-B" which binds excessively to C3b and induces an increased stability and activity of the C3 convertase, resistant to decay by CFH, with enhanced formation of C5b-9 complexes and deposition of C3-fragments at endothelial cell surfaces [81]. CFB-mutated patients exhibit a permanent activation of the alternative pathway with very low C3. Plasma CFB levels may be normal or low (Table 1 and Table 2). Mutations in *CFB *are rare, accounting for only 1-4% of aHUS patients [18,23,31,81,82]

#### C3 mutations

Heterozygous mutations in *C3 *were first described in 2008 in 13 patients from 11 families [83]. Most *C3 *mutations induce a defect of the ability of C3 to bind to regulatory protein MCP and are indirect gain of function mutations leading to an increased capacity for CFB to bind to C3b and an increased formation of the C3 convertase. Plasma C3 levels are low in 70-80% of patients (Table 1 and Table 2) [18,83,84].

*C*3 mutations account for 2 to 10% of aHUS patients (Table 3) [18,23,83]

#### Thrombomodulin mutations

Recently, heterozygous mutations in *THBD *have been demonstrated in 13 patients from the Italian cohort [18,45]. *In vitro*, THBD binds to C3b and CFH and negatively regulates complement by accelerating CFI-mediated inactivation of C3b in the presence of cofactors CFH or C4b- binding protein. The authors could demonstrate that the THBD variants were less effective than wild-type THBD in enhancing CFI-mediated inactivation of C3b. Cells expressing mutant THBD have a reduced capacity to degrade C3b and to generate thrombin-activable fibrinolysis inhibitor that cleaves C3a and C5a. C3 levels are decreased in half of THBD-mutated patients (Table 1).

THBD mutations account for 3% and 5% of aHUS patients in the USA [23] and Italian [18,45] registries respectively.

#### Combined mutations

Up to 12% of aHUS patients have various combinations of 2 or more mutations of *CFH*, *CFI, MCP*, *C3, CFB or THBD *[18,23,28,31].

#### In conclusion

One or several abnormalities of the complement system are presently demonstrated in 70% of children and adults with aHUS, and 30% of aHUS remain unexplained today. Not unexpectedly, the percentage of patients within the various subgroups shows some variations over the years according to countries and registries.

### Familial aHUS, incomplete penetrance and genetic variability

A familial occurrence of the disease is observed in approximately 20% of pedigrees [17,18]. In familial aHUS, the disease has an autosomal recessive or dominant pattern of inheritance. The absence of familial history of HUS does not preclude the possibility of a genetic transmission of the disease. Familial aHUS is observed in patients with complement mutations but also in the unexplained group.

**Figure 5:**
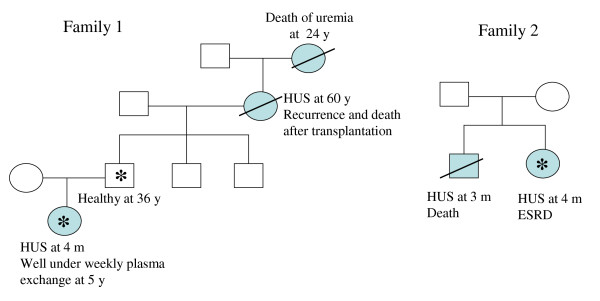
**Mode of transmission and intrafamilial phenotype variability of atypical hemolytic uremic syndrome: example from two families with heterozygous *CFH *mutation**. *CFH *mutation: W1183R, SCR 20 (Family 1); W1183L, SCR 20 (Family 2). Notice i) the autosomal dominant (Family 1) or recessive (Family 2) mode of inheritance of the disease ii) the intrafamilial phenotype variability and incomplete penetrance in Family 1. Affected individuals are indicated with filled symbols. Deceased individuals are crossed. Carriers of the *CFH *mutation are indicated by an asterisk. Courtesy of Professor G. Deschênes (Hôpital Robert Debré, Paris), with permission.

The majority of complement mutations are heterozygous in aHUS patients. *De novo *mutations are exceptional and the same mutation is almost constantly present in one parent -generally healthy-of the propositus [18]. Penetrance of complement-aHUS has been found to be only approximately 50% as half of the family members who carry the mutation do not present the disease by age 45 [20,50]. This has been observed for all mutations, i.e. *CFH, MCP, CFI *[18,38,46,85], *CFB *[80], *C3 *[18,83,84] and *THBD *[18,45]. The identified mutation therefore appears as a risk factor to the disease rather than its direct and unique cause. In addition, age at onset and severity of the disease may vary among family members with the same mutation (Figure 5). The role of various polymorphisms as independent or additional susceptibility factors to aHUS has been demonstrated in the genes encoding CFH [18,26,85-87], MCP [26,86], CFHR1 [88] or C4b-BP [89]. These sequence variations in the human genome are mostly changes of a single base (Single Nucleotide Polymorphism, SNP), which can be associated with a change of amino-acid in the protein, inducing a partial gain or loss of function. More than 30 SNPs are localized in the RCA locus. For instance, the frequency of an haplotype of *CFH *(*CFH *gtgt), defined by 4 SNPs localized in SCR 1, 7, 11 and 16 and one haplotype of *MCP *(*MCP *gggac) defined by 5 SNPs localised in the gene promoter and the intronic MCP gene, was significantly increased in patients compared to normal controls. In some families, it appeared that the proband had inherited the complement mutation from one parent, and an allele carrying the polymorphism of *CFH *and/or *MCP *from the other parent, while the healthy mutation carriers did not inherit the aHUS-associated *CFH *and *MCP *polymorphisms [26,85,86]. However, even when an unfavourable group of risk factors co-segregates, the disease may not manifest until middle age, suggesting that a trigger (such as infection or pregnancy) hypothetized to be an endothelial cell insult, is required to initiate the disease in individuals unable to control complement activation. aHUS therefore appears as a multifactorial disease resulting from environmental events that initiate endothelial damage and genetic factors (mutations and at risk polymorphisms) that determine progression of the disease.

**Figure 6:**
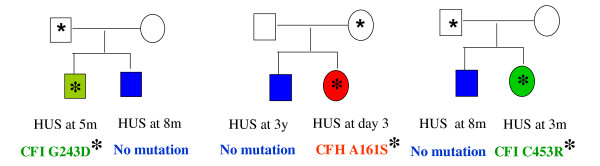
**Unknown risk factor(s) to atypical hemolytic uremic syndrome can be associated with identified mutations: example from three families**. In the 3 families, one child with aHUS has a mutation in CFH or CFI while a sibling also with aHUS has no mutation identified. Therefore the two siblings in each family share at least one unidentified risk factor. Affected individuals are indicated by filled symbols. Carriers of mutations are indicated by an asterisk. Courtesy of Professors R. Salomon (Hôpital Necker, Paris), E. Bérard (Hôpital de l'Archet, Nice) and G. Deschênes (Hôpital Robert Debré, Paris), with permission.

In practice, it is impossible to forecast the risk of occurrence of HUS in family members presenting the same mutation as their proband. Another problem is that several genetic anomalies may be present in one family, some of them unknown. For example, in three families from the French Pediatric Registry, one child with aHUS had *CFH *or *CFI *mutation respectively, while his sibling also with aHUS had no mutation [17 + unpublished data] (Figure 6). This shows that other unidentified genetic risk factors may be present in the patients and healthy family members.

### Genotype-phenotype correlations

Age at onset is similar in adults whatever the complement anomaly. On the opposite, it varies in children according to complement anomaly [17,18]. In the French pediatric cohort, very young age at onset was predominantly observed in patients with *CFH *(median 6 months, from 3 days to 3.6 years) or *CFI *(median 2 months, from 1 day to 3.8 years) mutations, while onset before the age of one year was not observed in children with *MCP *mutations (median 4.6 years, from 1.6 to 11.3 years [17]. In patients from the Italian registry, the earliest onset (between birth and 1 year) was in children with *CFH*, *C3 *or *THBD *mutations [18]. Thus, the majority of children with *CFH*, *CFI*, *C3 *and *THBD *mutation start the disease before 5 years of age. On the opposite, most children with anti-CFH antibodies (median age 8.5 years, from 8 months to 14 years, most frequently 7 to 11 years) or *MCP *mutations, and a few with *THBD *mutations, start the disease during late childhood or adolescence (Table 3) [17,18,37]. Outcome and prognosis according to complement abnormality are indicated in Sections Outcome and Prognosis.

## Diagnostic methods

Methods for complement investigation are indicated in Table 4 and Table 5. Except for the concentration of plasma C3 and C4, investigations of the complement system require specialized laboratories [61]. The list of laboratories providing specialized investigations of the complement system is available in references [38,90]. Except for the study of MCP expression on peripheral leucocytes and the screening for mutations, blood samples must be collected before plasma infusion (PI) or plasma exchange (PE). Normal range of each protein has to be determined for each technique in each laboratory using 100 healthy donors of the same ethnicity as the patients. A major issue is the lack of international standards for CFH and CFI. In addition to physiological variability of plasma CFH concentration, the dosage of CFH is particularly problematic, explaining variations of results between laboratories [61]. Assessment of plasma C3, C4, CFH, CFI and CFB levels, membrane expression of MCP on blood leucocytes, and screening for anti-CFH antibodies is mandatory and results should be available as soon as possible.

**Table 4:** Methods for assessment of plasma and membrane complement proteins and screening for anti- factor H antibodies

Plasma or membrane complement Proteins	Plasma concentration (mg/L) (- 2 to + 2 SD) or membrane expression	Technique	Laboratory	Interpretation
**C3**	660-1250	Nephelometry	Basic complement screen	Severe complement consumption through the alternative pathway indicated by very low plasma levels of C3 and CFB. Frequently, there is only an isolated moderate decrease of C3 level with normal CFB level
	
**CFB**	93-380	Nephelometry	Specialized diagnostic	

**CFH**	330-680 (no international standard)	ELISA	Specialized diagnostic	CFH or CFI less than 60% of normal are compatible with quantitative deficiency
	
**CFI**	40-80 (no international standard)	ELISA (or radial immunodiffusion)	Specialized diagnostic	

**Anti-CFH Ab**	Screening	ELISA	Specialized diagnostic	The title is expressed in Arbitrary Units (AU)

**MCP**	Mean fluorescent intensity (MFI)	FACS ^(a) ^with anti- MCP phycoerythrin -conjugated antibodies	Specialized diagnostic	No MCP expression is detected in patients with homozygous MCP deficiency. The MFI in patients of heterozygous MCP deficiency is around 50% of the normal range

(a) Usually performed on peripheral granulocytes or mononuclear cells in EDTA-blood sample.

Elisa, enzyme-linked immunosorbent assay; EDTA, ethylene diamine tetra-acetic acid; FACS, fluorescence-activated cell sorter.

**Table 5:** Genetic screening of the complement system

Gene	Location	Method of choice for mutation screening	Number of exons
***CFH***	RCA, Chr 1q32	Direct sequencing analysis	22

***CFI***	Chr 4q25	Direct sequencing analysis	13

***MCP***	RCA, Chr 1q32	Direct sequencing analysis	14

***C3***	Chr 19p13.3	Direct sequencing analysis	42

***CFB***	Chr 6p21.3	Direct sequencing analysis	18

***THBD***	Chr 20p11.2	Direct sequencing analysis	1

Chr, chromosome; CFH, factor H; CFHR1, factor H-related protein 1; CFI, factor I; MCP, membrane cofactor protein; CFB, factor B; THBD, thrombomodulin.

**Figure 7:**
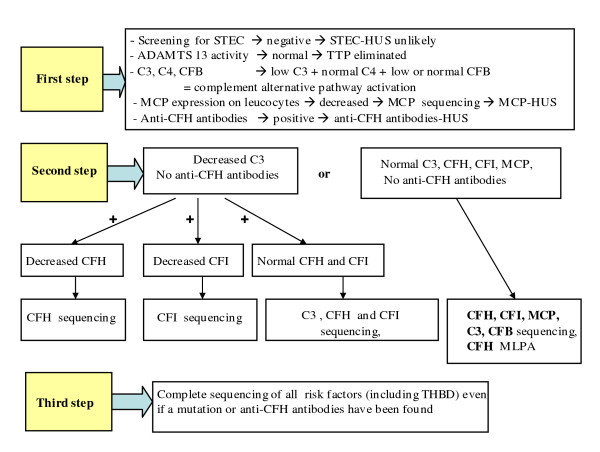
**Complement system screening strategy in atypical hemolytic uremic syndrome**. Knowledge of complement proteins plasma concentrations guides the investigator for the choice of which gene to study first and for the validation of genetic screening. Of note i. C3 may be low despite normal CFH or CFI plasma levels in patients with *CFH *or *CFI *mutations respectively. ii. C3 and CFH plasma levels are normal in patients with hybrid *CFH *detected by MLPA. STEC: Shiga-toxin producing *Escherichia coli*; ADAMTS 13, A Desintegrin And Metalloproteinase with a ThromboSpondin type 1 motif, member 13; CFH: factor H; CFI: factor I; CFB: factor B; MCP: membrane cofactor protein; THBD: thrombomodulin.; MLPA, multiplex ligation dependent probe amplification.

In children, age at onset guides genetic investigations (Table 6): in patients with onset before the age of one year, *CFH*, *CFI *and *C3 *should be screened first, whether C3 plasma concentration is decreased or not. If onset is after one year of age and C3 concentration is normal, *MCP *mutation should be first investigated. As anti-CFH antibodies-HUS predominates after the age of approximately 7 years and in pre-adolescents and adolescents, screening for anti-CFH antibodies is a priority at this age, especially if C3 concentration is decreased. Screening for *CFB *and *THBD *mutation is necessary in case no mutation is found in *CFH*, *CFI*, *MCP *and *C3*, whatever the age of onset. Figure 7 shows genetic screening strategy according to plasma levels of C3, CFH and CFI and expression of MCP. This strategy is theoretically valid, although not very realistic in practice.

**Table 6:** Atypical hemolytic uremic syndrome in children: age at onset and plasma C3 concentration as indicators of complement anomaly to screen in priority

Age at onset	Complement abnormality to screen first
Birth to < 12 months ± decreased C3	*CFH *, *CFI, C3 *mutation

> 1 year + normal C3	MCP decreased expression/mutation

> 1 year + decreased C3	*CFH, CFI, C3 *mutation

7-11 years ± decreased C3	Anti-CFH antibodies

CFH: factor H; CFI: factor I; MCP: membrane cofactor protein

Several messages are important: i. The association of low C3 and normal C4 plasma levels signs complement alternative pathway activation in the fluid phase. Very low C3 and CFB levels are indicative of intense alternative pathway activation, mildly decreased C3 with normal CFB is indicative of mild activation in the fluid phase. ii. Normal C3 and CFB levels do not eliminate a complement abnormality with dysregulated activation at the cell surface. iii. The assessment of complement proteins plasma level is insufficient and genetic analyses are necessary in any patient with aHUS, even if the plasma level of C3, CFH, CFI and CFB and the expression of MCP are normal. iv. However, knowledge of complement proteins plasma levels guides the investigator towards which gene to screen first (Figure 7) and helps him for the validation of genetic screening. For instance, if CFI level is low, and non *CFI *mutation is found, results of sequencing are re-examined and if necessary, sequencing repeated to find out the missed mutation; if C3 is low with normal CFH anf CFI levels and no anti-CFH antibodies, a C3 and then a CFB mutation must be looked for; if a patient has *MCP *mutation with low C3, a mutation in another factor than MCP must be looked for. v. C3 and CFH plasma levels are normal in patients with hybrid *CFH *detected only by MLPA, a technique now indicated for all unexplained aHUS. vi. As mutations have been identified everywhere in the various genes, screening of all exons is justified. vii. Since at least 10% of patients have mutations in two or more complement regulators, and some have a mutation in addition to anti-CFH antibodies, the identification of a mutation or anti-CFH antibodies does not preclude the necessity to study all genes, although the specific role of each complement abnormality on the outcome of the disease is unclear. viii. Screening for mutations and anti-CFH antibodies is mandatory before transplantation, especially in historical patients considered for transplantation (see Section Transplantation). ix. The functional consequences of each genetic abnormality should be determined in vitro by mutagenesis, if not already established [61]. Interaction between the physician and the referent person in the specialized laboratory about the pathogenicity (proven/most probable/unlikely/unknown) of the mutation is fruitful and should be encouraged.

## Differential Diagnosis

**Figure 8:**
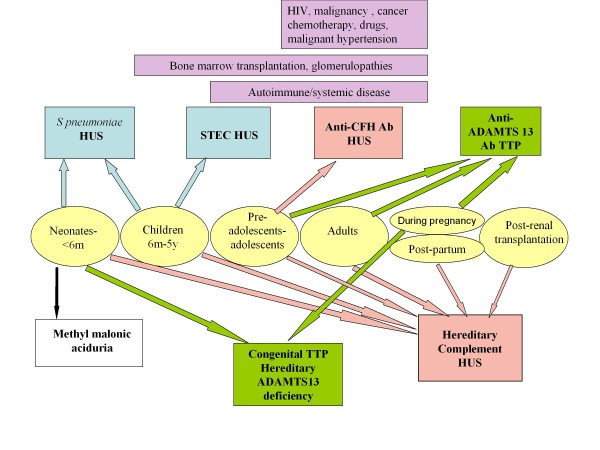
**The various subgroups of hemolytic uremic syndrome and thrombotic thrombocytopenic purpura according to age at onset**. Pink arrows and boxes: complement-HUS; green arrows and boxes: TTP; upper line: immune HUS and TTP; lower line: hereditary HUS and TTP. The figure also shows the 2 main infection-induced HUS (blue arrows and boxes) and the various causes of secondary atypical HUS (violet boxes), according to age. HUS: hemolytic uremic syndrome; TTP: thrombotic thrombocytopenic purpura; HIV: human immunodeficiency virus; STEC: Shiga-toxin producing *Escherichia coli*; ADAMTS 13, A Desintegrin And Metalloproteinase with a ThromboSpondin type 1 motif, member 13.

Complement-aHUS has to be differenciated from other forms of HUS and from TTP. Age at onset (Figure 8), family history, context and clinical presentation are generally indicative in children, while presentation may be more confusing in adults (Table 7). Biological confirmation is now available for the majority of patients [90,91].

**Table 7:** Clinical presentation of the various subgroups of hemolytic uremic syndrome and thrombotic thrombocytopenic purpura and investigations to confirm diagnosis

Age at onset and clinical presentation	Probable diagnosis	Investigations to confirm diagnosis
**Neonatal period** Severe jaundice Porto colour urine without major hematuria Consanguineous family and/or similar symptoms or neonatal death in siblings	Congenital TTP (Upshaw-Schulman syndrome)	ADAMTS 13 deficiency (< 10%) without anti-ADAMTS 13 antibobies Mutation in *ADAMTS13 *(autosomal recessive)

**Neonatal period-< 6 months** Failure to thrive, feeding difficulties, hypotonia ± developmental delay Consanguineous family	Methyl-malonic aciduria-associated HUS	Hyperhomocysteinemia, hypomethioninemia, methyl-malonic aciduria Mutation in *MMACHC *(autosomal recessive)

**< 2 years** Fever Invasive *S.pneumoniae *infection (proven or suspected): pneumonia, meningitis, septicaemia, especially if empyema or subdural collection	HUS due to *Streptococcus pneumoniae*	False positive Coombs test Positive cultures (blood, CSF) or PCR Positive T-activation test (exposure of the Thomsen-Friedenreich antigen on red blood cells) supports the diagnosis

**> 6 months-5 years** Diarrhea ± melena during the last 2 weeks Endemic region of STEC or *Shigella dysenteriae *infection	STEC-HUS *(Shigella dysenteriae-*HUS in endemic regions)	Stool or rectal swab: culture for STEC (Mac Conkey for 0157:H7); PCR for Stx Serum: anti-LPS antibodies against the most common serotypes in the local country

**Adolescents and adults** Fever Central nervous system manifestations No or mild renal involvement Autoimmune context (SLE, APLS, thyroiditis)	Immune TTP	ADAMTS 13 deficiency (< 10%) with anti-ADAMTS13 antibodies

**From birth to adolescence and adult age** No prodromic diarrhea or prodromic diarrhea but any of the following: - age < 6 months or > 5 years - insidious onset - relapse of HUS - suspicion of previous HUS - previous unexplained HUS - post-transplant HUS - pregnancy (post-partum) HUS - non synchronous familial HUS	Complement-aHUS	Complete investigation of the complement system

HUS, hemolytic uremic syndrome; TTP: thrombotic thrombocytopenic purpura; ADAMTS13: A Desintegrin And Metalloproteinase with a ThromboSpondin type 1 motif, member 13; MMACHC: methylmalonic aciduria and homocystinuria; CSF: cerebro-spinal fluid; PCR: polymerase chain reaction; STEC: Shiga-toxin producing *Escherichia coli*; Stx: Shiga-like toxin; LPS: lipopolysaccharide

In neonates and children less than 6 months of age, hereditary complement-HUS is the first line diagnosis, but pneumococcal HUS also needs urgent recognition and treatment [92-94], while hereditary congenital TTP and methylmalonic aciduria, both exceptional diseases, are alternative diagnoses that need specific investigations and treatment. In children from 6 months to 5 years of age, again the diagnosis of pneumococcal HUS must not be delayed. Post-diarrheal STEC-HUS largely predominates in this age group, but complement-HUS comes next. Preadolescents and adolescents mostly have complement-HUS, predominantly MCP-HUS and anti-CFH antibodies -HUS. Interestingly, it is also the age for acquired TTP due to anti-ADAMTS13 antibodies [95] (Figure 8). In adults, secondary aHUS or immune TTP may have common causes such as autoimmune diseases (systemic lupus erythematosus (SLE) or antiphospholipid (APL) antibodies syndrome) (Figure 8). Pregnancy may trigger both HUS and TTP, although pregnancy-TTP mostly occurs during the second and third trimesters while pregnancy-HUS is mostly a disease of the post-partum [39]. TTP and aHUS generally have different clinical presentation, with predominant neurologic involvement in TTP and renal involvement in HUS, but symptoms may overlap and complement-HUS has to be suspected whenever ADAMTS13 deficiency is not confirmed in a TTP- patient.

### Patients identified as having aHUS require full biological investigation (Table 8)

i. Investigations *for STEC infection at onset of HUS are required in all patients, including those identified as having aHUS*, as unusual presentation of STEC-HUS (unusual age or absence of diarrhea) can occur.

ii. *ADAMTS13 activity determination is required in all patients identified as having aHUS*, as manifestations of aHUS and TTP may overlap. In addition, the association of *CFH *mutation with a hereditary complete deficiency of ADAMTS13 has been reported [96]. Blood must be collected before PI or PE. Only ADAMTS13 activity below 10% of normal is significant of TTP. ADAMTS13 plasma concentration can now be determined within less than 24 hours by Elisa technic [97]. Laboratories for ADAMTS13 determination are indicated in [38,90].

iii. *Screening for defective cobalamine metabolism (homocystinuria with methylmalonic aciduria) is mandatory in all children with aHUS*. In the neonatal form of this exceptional intra cellular vitamin B12 metabolism anomaly, mortality is extremely high once HUS has developed, because of multivisceral failure [12,14]. The recommendation to perform diagnostic investigations for this disease in all children with aHUS comes from the report of a few cases of mild methylmalonic aciduria without neurological involvement, revealed by aHUS during late childhood. Such cases appear to have a favourable outcome both of HUS and the metabolic disease under continuous B12 supplementation [13]. Of note, the association of methylmalonic aciduria with *MCP *[98] or *CFH *[99] mutation has been reported, once again outlining that aHUS is a multifactorial disease.

iv. *In adults with aHUS, HIV infection and autoimmune disease must systematically be investigated*.

v. *Women with HELLP or post-partum HUS, and patients with post-transplant HUS, require complement investigation*. While no complement dysfunction has been demonstrated in patients with HIV-aHUS or HUS after bone marrow transplantation, complement mutations have been demonstrated in 36% of women with HELLP syndrome [100], 86% of pregnancy-HUS [39] and 29% of *de novo *HUS after kidney transplantation [101]. Of note, complement mutations have also been demonstrated in 18% of patients with SLE and/or APL antibodies who develop preeclampsia and 8.5% of preeclampsia patients lacking autoimmune disease [102]. The frequency of preexisting complement anomalies in aHUS complicating malignant hypertension has not been investigated.

**Table 8:** Investigations recommended in patients identified as having atypical hemolytic uremic syndrome, adapted from [90]

	Investigations
1. STEC infection	Stool or rectal swab: culture for STEC (Mac Conkey for 0157:H7); PCR for Stx Serum: anti-LPS antibodies against the most common serotypes in the local country

2. Disorders of complement regulation	C3, C4 (plasma/serum) Factor H, Factor I, Factor B (plasma/serum) Anti-factor H autoantibodies MCP (surface expression on leucocytes (polynuclear or mononuclear leucocytes by FACS) Gene mutation analysis in factor H, factor I, MCP, C3, factor B

3. ADAMTS13 deficiency inherited or acquired classification	Plasma ADAMTS13 activity or dosage (Elisa) ± inhibitor

4. Cobalamin metabolism:methyl malonic aciduria	Plasma amino-acid chromatography (high homocysteine, low methionine); urine organic acid chromatography (methyl-malonic aciduria) ± mutation analysis in *MMACHC *gene

5. HIV	Serology

6. Pregnancy, HELLP syndrome	Pregancy test, liver enzymes. Investigate as in 2 and 3

7. Miscellaneous	Antinuclear antibody, lupus anticoagulant, anti-phospholipid antibodies

STEC: Shiga-toxin producing *Escherichia coli*; Stx: Shiga-like toxin; PCR: polymerase chain reaction; ADAMTS13: A Desintegrin And Metalloproteinase with a ThromboSpondin type 1 motif, member 13;HIV: human immunodeficiency virus; HELLP: Hemolysis, Elevated Liver enzymes, Low Platelet count; MCP: membrane cofactor protein; FACS: fluorescence-activated cell sorter; MMACHC: methylmalonic aciduria and homocystinuria;

## Genetic Counselling and Prenatal Diagnosis

*De novo *mutations are exceptional and, if investigated, one parent of the proband has the mutation. Proband's siblings and children have a 50% chance of inheriting the mutation. However, as penetrance of the disease is approximately 50% and age at onset and clinical severity often variable among individuals with the same mutation within a family, it is extremely difficult to predict the risk of developing the disease and the outcome in the individuals at risk. Genetic counseling must also take into account that HUS results from a panel of genetic risk factors, some known and some unknown. Identifying one factor does not eliminate the responsibility of another one, not known today. In other words, it is as difficult to be sure that an individual has no risk of HUS as to be sure he is at risk.

Despite these caveats, genetic counseling has to be offered to parents and young adults with the disease or at risk. Prenatal testing is possible. Identification of the mutation(s) is necessary before prenatal testing is proposed. In addition, the functional consequences of the mutation need to be established, to be sure the mutation is at high risk of being disease-causing. However, similar to what happens for healthy family members with the mutation, the risk for the fetus to develop aHUS after birth is extremely difficult to forecast, even for mutations with a high degree of pathogenicity. Pre implantation genetic diagnosis may be an option.

## Management Including Treatment

### Supportive treatment

Progress in intensive care and dialysis has contributed to the decrease of mortality, especially in young children. In practice, any patient suspected of having aHUS needs to be transfered to a specialized centre (Nephrology or if necessary Critical Care) where management of acute renal failure and hypertension, the various techniques of dialysis and plasma exchange (PE) are daily practice.

Although the scope of this review is not to develop the management of acute renal failure, some specificities need to be indicated: i. Platelets infusions are contra-indicated, as they might worsen the TMA process, unless the patient is bleeding (exceptional) or when a surgical procedure at risk of being hemorrhagic is scheduled in a severely thrombocytopenic patient (platelets < 30 000/mm^3^). ii. Vascular access most often rely on a central catheter allowing dialysis and PE: the vein (femoral, subclavian or internal jugular) depends on patient's age and local practice. Percutaneous insertion of double lumen catheter has to be performed by a trained physician. The protection of peripheral and central veins (no ligation) is of outmost importance in these patients who may need long term vascular access for hemodialysis or PE.

Considering the frequency of relapses triggered by infections, clinicians should be vigilant for signs of infections and treat them when appropriate. Whether vaccination might trigger HUS is scarcely documented. The benefit of vaccination, especially against influenza (seasonal and H1N1, using the vaccine without adjuvant), most probably outweighs its risk.

### Plasmatherapy

Plasmatherapy remained the first line treatment of aHUS until 2010, based on expert opinion rather than clinical trials [31,90,91,103]. No prospective trial has been done. Poor renal outcome in retrospective registries [17,18] could be related to delayed, insufficient or too short plasmatherapy. Viroinactivated fresh frozen plasma (FFP) brings normal amount of CFH, CFI, CFB and C3. PE removes mutant CFH, CFI, CFB and C3, anti-CFH antibodies and other triggers of endothelial dysfunction and platelet hyperaggregability, while volume restitution with FFP brings the functional proteins. In addition, PE prevents volume overload and cardiac failure when large amounts of FFP are infused. It also prevents the hyperprotidemia which develops when high amounts of plasma are infused several times a week.

#### Plasmatherapy in patients with CFH mutation

Overall, 63% of patients with *CFH *mutation who received some form of plasmatherapy (plasma infusion or plasma exchange) in the Italian Registry had a response to plasma therapy (either complete or partial (hematological remission with renal sequel). However, the percentage of complete recovery under plasmatherapy was only 5% and evolution to death or end stage renal disease 37% [18]. Twelve observations, mainly in children, show that early intensive plasmatherapy can rescue HUS and long term plasmatherapy prevent relapses and evolution to end stage renal failure (ESRF) in *CFH*-mutated patients [17,103-115]. These patients received PE (40-60 ml/kg with FFP for restitution) or PI (10-15 ml/kg) at the acute phase, most of them daily during at least 5 days and up to 2 weeks, then tapered to long term maintenance plasmatherapy (PE or PI from weekly to every 2 to 4 weeks). Most of them had relapses during infections, rescued by intensification of plasmatherapy. Ten of the twelve patients had preserved kidney function after 1 to 6 years follow-up under plasmatherapy, but two developed ESRF after 4 years [107] and 7 years [115], showing that it is uncertain whether the favourable effect of plasmatherapy can be maintained for decades. On the opposite, most patients who received plasmatherapy only during acute episodes died or were in ESRF within less than one year [103,113,116-118]. The response might be different according to genotype. A few observations suggest that PE could be superior to PI in patients with a dysfunctional mutant CFH (type 2 mutations), possibly because PE removes this dysfunctional CFH [113,114].

#### Plasmatherapy in patients with CFI mutation

Only 25% of patients with *CFI *mutation who received plasmatherapy in the Italian Registry had a response and 75% progressed to death or ESRF [18]. Five CFI-mutated patients (3 of them with associated risk factors) who received PE or PI [36,77,103,119] at the acute phase had complete or partial remission. All had relapses and all except one developed ESRF within a few weeks or months. A larger number of patients would be necessary to document the effect of plasmatherapy in patients with *CFI *mutations and the role of associated mutations in the response to plasma therapy.

#### Plasmatherapy in patients with MCP mutation

As MCP is not a circulating protein, a beneficial effect of plasmatherapy is unlikely to be expected in *MCP*-mutated patients. At least ninety per cent of patients undergo remission from acute episodes, whether or not they receive plasmatherapy [17,18,21]. However, PE is often performed during flares of HUS, to clear up noxious factors such as aggregating factors/triggers of endothelial lesions. Long-term PE does not seem to be of benefit in MCP-HUS [120].

#### Plasmatherapy in patients with C3, CFB or THBD mutation

The benefit of plasmatherapy is scarcely documented in these subgroups. Fivety seven per cent of C3-mutated patients and 88% of THBD-mutated patients who received plasmatherapy in the Italian Registry had a response (either complete or partial remission (hematological remission with renal sequel), and 43% and 13% respectively progressed to death or ESRF [18]. Remission with PE or PI has been reported in 2 patients with C3 mutation [84,121] and 3 with CFB mutation [80-82].

#### Plasmatherapy and immunosuppressive drugs in aHUS with anti-CFH antibodies

PE, to remove the antibodies, is first line treatment in anti-CFH antibodies-HUS. However, antibodies titer often reincreases after PE cessation and relapses of HUS frequently occur. Therefore the association of immunosuppressive treatment is recommended, using steroids and azathioprine, mycophenolate mofetil, intravenous cyclophosphamide or anti-CD20 [37,68,72,122-124]. The duration of plasmatherapy and the choice of the immunosuppressive drug are not presently standardized. Both should be guided by the evolution of anti-CFH antibodies titer. High antibody titer is correlated with the risk of relapses, which in turn increase the risk of renal sequels [37].

#### Recommendations for the practice

Since complement and ADAMTS13 information is usually not available when the patient presents with symptoms of aHUS, expert opinion recommends empiric plasmatherapy to be started as early as possible, within 24 h of presentation [90,91,125]. First line treatment should be PE, with exchange of 1.5 plasma volume (60-75 ml/kg) per session, replaced by FFP. When PE cannot be performed within 24 h of presentation, PI of 10-20 ml/kg should be given if the patient is not volume overloaded and/or hypertensive and does not show symptoms of cardiac failure.

PE should be performed daily until platelet count, LDH and hemoglobin levels are normalized and renal function clearly improving since several days. Persistence of hemolysis or lack of improvement of renal function after 3-5 daily PE have to be regarded as criterium for uncontrolled TMA even if platelet count has normalized (a fortiori if thrombocytopenia persists), and as an indication to maintain daily PE or, in recent days, to switch the patient to eculizumab (See section Complement inhibitors: the new treatment in 2010-2011). When disease activity is controlled by daily PE, recommended subsequent frequency of PE/PI is five times a week for 2 weeks and three times a week for the subsequent 2 weeks. Further frequency has to be decided on a case by case basis, according to evolution and to which risk factor is demonstrated. The demonstration of *MCP *mutation allows the withdrawal of plasmatherapy. For patients with *CFH*, *CFI*, *C3 *or *CFB *mutations, modality (PE or PI) and interval of time between sessions has to be determined individually. Plasmatherapy should probably never be stopped in patients with a *CFH *mutation, maybe also a *CFI *mutation combined with another complement anomaly, or a *C3 *or *CFB *mutation. However, an attempt to withdraw plasmatherapy is generally considered in patients who do not present any manifestation of HUS despite tapering PE/PI to monthly or less than monthly administration since several months or years. In case of infection, biological controls should be performed to detect and treat a potential relapse early, by intensification of plasmatherapy to daily sessions.

#### The limits of plasmatherapy

Logistic and technical difficulties may limit the feasibility of PE over the long term. PE need technically trained center, especially for children [126,127]. If the patient has no arteriovenous fistula, a central venous catheter similar to those used for hemodialysis is necessary, with the risk of central venous thrombosis, particularly in young children, and infection. Some patients develop anaphylactic reactions to FFP, which may require cessation of any form of plasmatherapy.

### Renal transplantation: indications, risks and new issues

#### The risk of post-transplant recurrence of aHUS according to complement abnormality

Any aHUS patient who has reached ESRF is theoretically a candidate to renal transplantation. However, the overall risk of aHUS recurrence after renal transplantation is 50% and the risk of graft loss 80-90% in patients with recurrence [18,128-131] (Table 3). A high risk of graft failure due to arterial thrombosis has also been reported in children [17]. The risk of post-transplant aHUS recurrence is 75-90% in patients with *CFH *mutation, 45-80% in patients with *CFI *mutation, 40-70% in patients with *C3 *mutation [83,131]. The 3 patients with *CFB *mutations [80,81] and one with *THBD *mutation [45] who were transplanted all lost the graft to recurrence. On the opposite, the risk of recurrence is low (from zero [18] to 15-20% [131] in patients with *MCP *mutation, a logic issue as the graft brings the non-mutated MCP protein. The variability of the risk of post-transplant recurrence for instance in the CFI, C3 and MCP-mutated groups has to be interpreted with the present knowledge of a high frequency of associated genetic risk factors. As more than half of CFI-mutation carriers have been shown to harbor an additional susceptibility factor for aHUS, the risk of recurrence in patients with isolated CFI mutations should be reassessed [28]. In the same line, the few MCP-mutated patients reported with post-transplant recurrence could have had uninvestigated associated complement abnormalities responsible for the recurrence.

Post-transplant recurrence generally occurs very early in patients with *CFH *mutation: during the first days or the first month in half of reported cases, between the second and the sixth month in the other cases (data issued from references in [129]). However, some patients have recurrence only after several years. The delay until recurrence seems more variable in patients with *CFI *mutation, either during the first days post-transplant or after several months or years [129,132]. Recurrence sometimes does not present as a full-blown HUS, but as heavy proteinuria and a progressive deterioration of the graft function without hemolysis and thrombocytopenia, with TMA lesions at graft biopsy. In the majority of cases, untreated recurrence ends-up in graft loss, typically within the first year of transplantation.

The risk of post-transplant recurrence in patients with anti-CFH antibodies is not well documented, but recurrence can be expected if a high titer of antibodies persists at the time of transplantation. A reduction of anti-CFH antibody levels with plasma exchanges and rituximab enabled successful transplantation in 2 patients [37,122,133]. Publications have reported that recurrence-free transplantation was achievable in patients with anti-CFH antibody without any specific treatment [71,134]. However, pretransplant screening of anti-CFH antibodies or determination of their titre was not available in these observations. The assessment of the risk of post-transplant recurrence is complicated by the report that patients with anti-CFH antibodies may also carry mutations in *CFH, CFI, MCP *or *C3 *[71].

There is also a risk of recurrence in patients with no mutation and no anti-CFH antibodies, although not well established.

#### Living-related kidney donation is not recommended

Considering the risk of graft loss to recurrence, living-related kidney donation must be considered as contra-indicated for patients with *CFH*, *CFI*, *CFB*, *C3 or THBD *mutation, questionable for patients with unexplained aHUS, debatable for patients with *MCP *mutation. In addition, the risk for the donor to develop HUS after kidney donation has to be taken into account. This has been reported in four donors who had HUS three weeks to ten months after donation [129]. *CFH *mutation was subsequently demonstrated in one of the donors and his recipient. Considering the incomplete penetrance of the disease, the role of complement gene polymorphisms and the genetic variability within members of a single family, it is impossible to reach one hundred percent certitude of "no risk of aHUS" for living-related donors.

#### Prevention/rescue of post-kidney transplant aHUS recurrence

*Bilateral nephrectomy *of native kidneys is often performed before transplantation because of severe hypertension or ongoing hemolysis and thrombocytopenia. Unfortunately, this does not reduce the risk of aHUS post-transplant recurrence [17].

#### Avoidance of calcineurin inhibitors

Some studies have suggested that avoidance of calcineurin inhibitors, that have an endothelial toxicity, might be beneficial [135] but others have reported conflicting results [128,129,131]. One explanation could be that calcineurin-free protocoles often rely on sirolimus, now demonstrated to be toxic to the endothelium and predisposing to TMA, through its role of VEGF down-regulation [7-11]. Owing to these problems and the increased risk of rejection in calcineurin-free regimens, aHUS is not considered *per se *as a contraindication to calcineurin inhibitors [125].

#### Plasmatherapy to rescue or prevent post- kidney transplant aHUS recurrence

**Figure 9:**
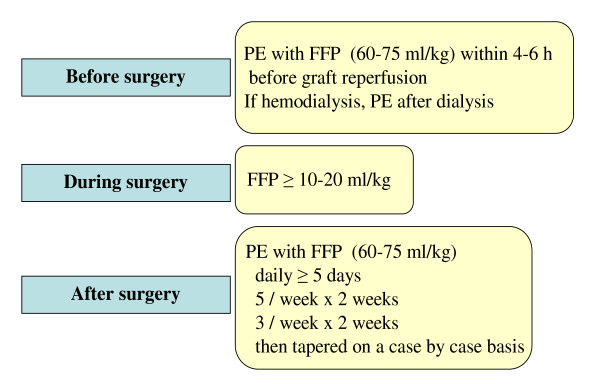
**Recommendations for plasmatherapy to prevent post- kidney transplant recurrence of hemolytic uremic syndrome, according to the Consensus Study Group **[125]. Of note, preventive eculizumab (started before transplantation) now has to be considered for patients at very high risk of recurrence. PE, plasma exchange; FFP, fresh frozen plasma.

Many patients of historical series have received some form of plasmatherapy at the time of recurrence [17,128-131]. As time at plasmatherapy initiation, modalities (PE or PI), volume of FFP infused or exchanged, frequency and duration were highly variable, the effect of plasmatherapy is difficult to ascertain. However, most studies suggest that plasmatherapy at the time of recurrence often fails to rescue kidney function and to prevent graft loss [128-131]. Therefore preventive plasmatherapy is now recommended [125]. In the line of plasmatherapy recommended for aHUS on native kidneys, one PE session should be performed just before transplant surgery, FFP infused during surgery, and PE continued daily for at least 5 days, then 5 sessions per week during 2 weeks, 3 sessions per week during 2 weeks, subsequently tapered on a case by case basis according to the evolution (Figure 9). To our knowledge, preventive plasmatherapy has been successful to prevent recurrence in ten renal transplant recipients, including 5 with *CFH *mutation [113,131,136-138], 3 with *CFI *mutation [131,139], 2 with *C3 *mutation [121,131]. The efficiency of prophylactic plasmatherapy started before renal transplantation is particularly demonstrated in one family [113,137]. Three siblings, including two identical twins, had aHUS with *CFH *S1191L mutation, SCR20. The eldest child lost a first graft for recurrent HUS. One of the identical twins had preserved function of her native kidneys under prophylactic PE at 6 years follow-up. The second twin had a successful renal transplantation with PE started just before surgery and maintained subsequently, with serum creatinine 127 μmol/l at 5 years post-transplantation. The eldest sister lost a second graft despite prophylactic PE, when they were decreased from 1/week to 1/two weeks [113]. A third graft was successful under 2 PE weekly, but the child developed plasma intolerance and was switched to eculizumab [137]. Of note, two other patients, both with a *CFH *R1210C mutation in SCR20, received pre, per and postoperative PE/PI for only 2.5 and 3 months respectively and were free of recurrence at 1 year and 3 years follow-up respectively [136,138]. These observations confirm the benefit of preventive plasmatherapy started before surgery and maintained intensively during the first months after transplantation. They also show that subsequent program of plasmatherapy can only be empirical.

#### Combined liver-kidney transplantation to cure aHUS

As CFH, CFI, CFB and C3 are synthesized in the liver, liver or combined liver-kidney transplantation was logically proposed. To our knowledge, there have been 20 such transplants (19 for CFH mutation, 1 for CFB mutation) [51,125,140-147] (10 unpublished cases). Most (18) of the patients were in ESRF and received combined liver-kidney transplantation. The initial experience in 3 children with *CFH *mutation was disappointing, as 2 patients had primary hepatic non-function, with extensive microvascular thrombosis and complement deposition [140,141]. One of them recovered after a second liver transplantation and had no symptoms of HUS for 3 years, showing that liver transplantation did cure HUS, but ultimately died from the neurologic sequel of the hepatic encephalopathy [125,140]. The other child died in the early post-transplant period [141]. The third child had no symptoms of HUS during nearly one year after isolated liver transplantation, again confirming the efficiency of the procedure, but died from post-transplant lymphoproliferative disease [142]. The first 2 observations suggested that it was necessary to provide a large amount of CFH just before liver reperfusion, to obviate the massive complement activation associated with reperfusion and its thrombogenic consequences. Therefore, four subsequent combined liver-kidney transplantations, also in children with *CFH *mutation, were performed with intensive pre-operative plasmatherapy (PE with FFP 50-100 ml/kg just before surgery, FFP infusion (20-35 ml/kg during surgery or PE between liver and kidney transplantation), associated with post-operative anticoagulation. Both grafts had normal function at follow-up now largely over one year, and HUS was cured [143-145]. Following this positive experience, recommendations for intensive pre and per operative plasmatherapy to obviate complement activation during liver reperfusion were published [125]. Of the 14 combined transplantations performed with preconditioning PE and plasma infusions, 12 have been successful. However, 2 children died from operative vascular difficulties (hepatic artery thrombosis in one, cerebral ischemia related to superior vena cava syndrome during manipulation of the inferior vena cava in the other) [personal communication from J.Saland, with permission], indicating a 14% operative death rate in patients receiving combined liver-kidney transplantation under plasma therapy. Therefore, the decision of combined transplantation to cure the disease needs a precise appreciation of the risks/benefits on an individual basis.

### Complement inhibitors: the new treatment in 2010-2011

#### Eculizumab

**Figure 10:**
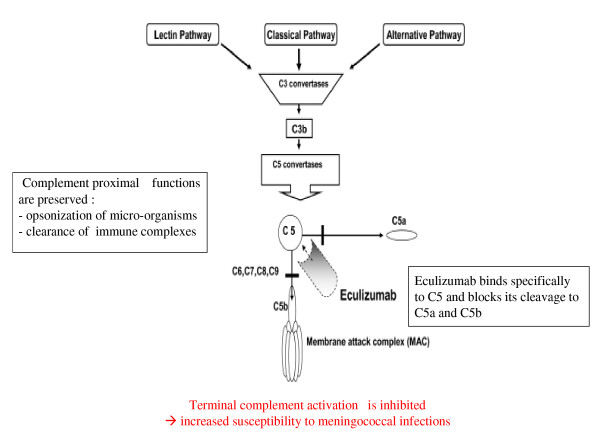
**Blockade of terminal complement activation, adapted from **[149]. Eculizumab binds to C5 and prevents the formation of the membrane attack complex by reducing cleavage of C5 to C5a and C5b.

The activation of C5 is essential for the development of aHUS [55,148]. Eculizumab (Soliris^®^, Alexion Pharmaceuticals, Cheshire, CT, USA) is a recombinant, humanized, monoclonal immunoglobulin G antibody that targets C5. Eculizumab blocks the cleavage of C5 to C5b, ultimately preventing the generation of the proinflammatory peptide C5b and the cytotoxic membrane attack complex C5b-9 (Figure 10).

Eculizumab is approved worldwide for the treatment of paroxysmal nocturnal hemoglobinuria (PNH) and its efficacy and good tolerance has been demonstrated in several hundreds of patients with this disease, some of them treated since up to 10 years [149-151]. Eculizumab has been granted Orphan Medicinal Product Designation for the treatment of aHUS by the U.S. Food and Drug Administration in May 2009 and the European Medicines Agency in August 2009. This new treatment has been the object of eager expectations from patients and physicians during recent years [115,152,153]. 2009 and 2010 have seen the first treatments of aHUS patients with eculizumab.

Blockade of the complement terminal pathway induces an increased risk of *Neisseria meningitis *infection [154]. Therefore patients must receive vaccination against *Neisseria meningitis *before being treated with eculizumab. However, as no vaccine presently protects against the B serotype, patients and physicians have to be aware of symptoms that would necessitate urgent investigations and antibiotherapy. Some countries (such as France) require permanent antibioprophylaxis during eculizumab treatment in addition to vaccination.

Recommended doses for aHUS patients are 30% higher than for PNH, in order to completely block the complement terminal activation. Schedule of administration is the same as for PNH: in adults, 900 mg (intravenous over 30 minutes), weekly for 4 weeks (a total of 4 injections at weekly interval), then 1200 mg for the fifth injection and then every 14 days as maintenance treatment on the long term. These doses induce eculizumab circulating trough levels > 35 μg/mL that consistently block terminal complement activation. Doses for children are adapted to weight. As experience with eculizumab is limited in children, control of the pharmacokinetics and pharmacodynamics of the drug and the degree of complement blockade (CH50 < 10% expected) will be important in this age group.

#### Clinical experience with eculizumab in aHUS

At present, 17 patients have been published and reported in congresses (abstracts available on the net) who received eculizumab either for aHUS on their native kidneys (Table 9) [155-161] or to rescue [121,131,137,162-166] or prevent [167-169] post-transplant recurrence (Table 10). Of the 17 patients, 8 were children (aged from 19 months to 18 years) and 6 had CFH mutation, 2 had C3 mutation, 1 had CFI mutation, 4 had no mutation identified and genetic was not documented in 2. In the 7 patients treated for aHUS on their native kidneys (Table 9), improvement in platelet count, cessation of hemolysis and improvement of kidney function strikingly occurred within a few days after eculizumab initiation. All five patients maintained on long term eculizumab had preserved renal function at follow-up from 10 weeks to 2 years 4 months. Two patients who received a single dose of eculizumab went to ESRF after a relapse of HUS 1 month [157] and 2 months [158] after eculizumab cessation. In addition, one of them was treated late in the course of the disease, after approximately fifty days on dialysis [157]. Of the 10 patients treated to prevent or rescue post-transplant recurrence, 6 had lost one or two previous grafts because of recurrence (Table 10). Remission of HUS was obtained within a few days and the three patients treated prophylactically did not experience recurrence. All 8 patients maintained on long term eculizumab treatment had preserved graft function and remission of HUS at follow up from 4 months to 2 years 5 months. Two patients who received a single dose had subsequent relapse after 11 and 21 months and ultimately lost the graft [162,165].

**Table 9:** Case reports of patients with atypical hemolytic uremic syndrome on their native kidneys treated with eculizumab

Reference	Mutation	Age at onset of HUS, evolution and response to plasmatherapy	Age at eculizumab initiation	Response to plasmatherapy of HUS episode at eculizumab initiation	Serum creatinine level at eculizumab initiation	Delay of hematological and renal improvement after initiation of eculizumab	Delay until complete remission of HUS after initiation of eculizumab	Protocol	Evolution of HUS Last Screat (follow-up under eculizumab)
Gruppo *et al* 2009 [155] R. Gruppo PC	NI	< 8 days 3 relapses over 11 m, PI sensitive	19 m	PE resistant	265 μmol/L	2 days	10 days	Complete protocol	Remission 35 μmol/L (2 y 4 m)

Fremont *et al* 2009 [156]	CFH	4 y	4 y	PE partially sensitive	80 μmol/L	ND	2 weeks	Complete protocol	Remission 26 μmol/L (10 weeks)

Mache *et al* 2009 [157]	NI	17.8 y PE sensitive 3 relapses at PE tapering	17.11 y	PE resistant	690 μmol/L	~ 3 days (platelets increase)	5 days (hematologic remission)	Single dose	Relapse at 2w ESRF

Kose *et al* 2010 [158]	NI	18 y	18 y	PE resistant	~310 μmol/L	1 day	~ 7 days	Single dose	Relapse at 2m ESRF

Lapeyraque *et al *2010 [159] AL. Lapeyraque PC	CFH S1191L V1197A	7 m 11 relapses over 5.4 y, PE/PI sensitive	6 y	PI resistant	108 μmol/L	A few days	1 week	Complete protocol	Remission 44 μmol/L (1 y 3 m)

Prescott *et al* 2010 [160] HC. Prescott PC	CFI p.A258T	47 y PE sensitive Relapse 2 w after PE cessation	47 y	PE resistant	610 μmol/L	- 7 days (Screat decrease) - 49 days (platelets normalization)	~1.5 m	Complete protocol	Remission 230 μmol/L (7 m)

Ohanian et al 2011 [161]	ND	50 y	50 y	No plasmatherapy	600 μmol/L	- 4 days (LDH decrease) - 10 days (Screat decrease)	~ 1.5 m	Complete protocol	Remission 198 μmol/L (2.5 m)

PC: personal communication, with permission; Screat: serum creatinine; CFH: factor H; CFI: factor I; NI: none identified; ND: not documented; ESRF: end stage renal failure; PE: plasma exchange; PI: plasma infusion

**Table 10:** Case reports of patients with post-transplant recurrence of atypical hemolytic uremic syndrome treated with eculizumab

Reference	Gene	Previous transplantations	Age and post-tx course before eculizumab initiation	Delay from recurrence to eculizumab initiation	Screat at eculizumab **initiation **μmol/L	Delay until platelet increase/hemolysis resolution/Screat decrease under eculizumab	Protocol	Recurrence after eculizumab cessation (delay)	Evolution under eculizumab Last Screat (follow-up under eculizumab)
Nurnberger 2009 [162] J.Nurnberger PC	CFH Y475S	1st tx: recurrence at 5 w, PE resistant, graft loss	37 y, 2^nd ^tx Recurrence at 6 w PE resistant	5 days	132	2 days/6 days/24 h	Single dose	Likely but not biopsy proven (21 m). Graft loss.	/

Chatelet 2009 [121] 2010 [163] V.Chatelet PC	C3 R570Q	1st tx: recurrence at 5 m, graft loss after 2 y	43 y, 2^nd ^tx Recurrence at 3 y PE sensitive/dependent	14 m	320	A few days/a few days/~1 month	Complete protocol	NA	2 recurrences of hemolytic anemia when injections delayed by 6-8 days 230 μmol/L (2 y 5 m)

Legault 2009 [164]	ND	No	34 y, 1^st ^tx Recurrence at 1 m and 5 m, PE sensitive initially, then resistant	9 m	323	ND/ND/4 weeks	Complete protocol	NA	Remission 238 μmol/L (6 m)

Davin 2010 [137] JC. Davin PC	CFH S1191L	1^st ^tx: recurrence at day 3, graft loss 2^nd ^tx under prophylactic PE: recurrence at 10 w, graft loss	17 y, 3^rd ^tx Preventive PE Recurrence at 4 m, rescue (PE intensification) Plasma intolerance at 10 m	10 m	131	NA (in remission at eculizumab initiation)	Complete protocol	NA	Remission 130 μmol/L (1 y 10 m)

Larrea 2010 [165] M.Lozano PC	NI	No	22 y, 1^st ^tx Recurrence at day 12 PE resistant	9 days	415	36 h/36 h/3 days	Single dose	Yes (11.5 m) Eculizumab resumed	Remission 177 μmol/L (5 m) Graft loss after 2^nd ^eculizumab cessation (humoral rejection)

Zuber 2010 [131] J. Zuber PC	CFH	1^st ^tx: recurrence, graft loss	24 y, 2^nd ^tx Preventive PI/PE Recurrence at day 1 PE resistant	4 days	500	24 h/24 h/3 days	Complete protocol	NA	Remission 62 μmol/L (9 m)

Al-Akash 2010 [166] SI. Al-Akash PC	C3 R570W	1^st ^tx: recurrence at 4 y, graft loss 2^nd ^tx: recurrence at 2 m, graft loss	15 y, 3^rd ^tx Preventive PE Recurrence at 2 m, PE partially sensitive	~20 days	220	A few days	Complete protocol	NA	Remission 115 μmol/L (1 y 5 m)

Zimmerhakl 2010 [167] M.Riedl PC	CFH W1183C	No	10 y, 1^st ^tx Preventive PE	Preventive eculizumab	~ 45	NA	Complete protocol	NA	No recurrence 44 μmol/L (2 y 1 m)

Weitz 2011 [168]	CFH E1198 stop	No	7 y, 1^st ^tx	Preventive eculizumab	NA (dialysis)	NA	Complete protocol	NA	No recurrence Normal Screat (7 m)

Nester 2011 [169]	Hybrid CFH	No	12 y, 1^st ^tx Preventive PE	Preventive eculizumab	NA (dialysis)	NA	Complete protocol	NA	No recurrence 80 μmol/L (4 m)

Preventive: started before transplantation or before recurrence; PC: personal communication, with permission; Screatinine: serum creatinine; CFH: factor H; NI: none identified; Tx: transplantation; NI: none identified; ND: not documented; NA: not applicable; PE: plasma exchange

International multicenter prospective phase II trials have been conducted in 2009-2010 in adults and adolescents (≥ 12 years) with aHUS (primary disease or post-transplant recurrence), either plasma resistant (17 patients) or plasma sensitive (on chronic plasmatherapy) (20 patients), who were switched from plasmatherapy to eculizumab [170,171]. These trials confirmed that eculizumab inhibits the TMA process in aHUS patients, with reversal of thrombocytopenia and hemolysis and improvement of renal function, whether they were unresponsive to plasmatherapy or on chronic plasmatherapy before receiving eculizumab. Response to eculizumab was achieved with the first dose of eculizumab and improvement of renal function continued with sustained therapy. In both groups, no patient required TMA intervention (plasmatherapy or new dialysis) while on eculizumab. Eculizumab was well tolerated. This beneficial response was observed both in patients with or without identified complement mutation. A prospective trial in children aged from 1 month to 18 years and a new trial in adults have started at the end of 2010, including patients with primary HUS or post-transplant recurrence, whether receiving plasmatherapy or not [172].

Overall, these results demonstrate the potential for eculizumab treatment as the new standard of care for patients with aHUS. 2011 probably is a transition year. Many physicians, especially pediatricians because of the difficulty of PE in young children, consider that eculizumab can now be administered without previous plasmatherapy. Whatever the patient's age, resistance or incomplete response to plasmatherapy (defined by persistant thrombocytopenia and/or hemolytic anemia and/or the absence of improvement of renal function after 3 to 5 daily PE), occurrence of a relapse at plasmatherapy tapering or cessation, intolerance to plasma or vascular access difficulties are or will be indications to replace plasmatherapy by eculizumab when available and refunded.

This new therapeutic option also changes the strategy for kidney transplantation. Transplantation physicians now have to consider they should treat the patient with eculizumab in case of HUS recurrence despite preventive plasmatherapy. If the risk of recurrence is extremely high (previous graft lost to recurrence in the patient, a family member or a patient with the same mutation from series and registries), the logic of preventive eculizumab to prevent recurrence is hardly questionnable. Finally, the indications of combined kidney-liver transplantation -under plasmatherapy or eculizumab to block complement activation at liver reperfusion-today appear limited, although this possibility to cure the disease must not be discarded.

#### New therapies for the near future

Other complement blockers which block the complement activation at the endothelium surface without blocking it in the fluid phase will become available in the near future [173]. A human plasma-derived CFH concentrate, developed by the Laboratoire Français du Fractionnement et des Biotechnologies, received the European Orphan Drug designation in January 2007 and ought to be available for clinical trials in the near future. This CFH concentrate has been shown in *Cfh*-gene knockout mice -which develop plasma C3 deficiency and massive accumulation of C3 along the glomerular basement membrane-to result in rapid normalization of plasma C3 levels and resolution of the glomerular basement membrane C3 deposits [174]. Recombinant CFH might also become available in the future [175,176].

## Outcome

Data on outcome and prognosis rely mostly on historical series, including patients who received either no plasmatherapy or plasmatherapy modalities which would now be considered as inadequate (started too late, not aggressive enough (PI instead of PE), stopped too early). However these series illustrate the natural outcome of the disease or the best evolution possibly obtained with the plasmatherapy modalities commonly achieved and/or feasible.

Among the French pediatric cohort, mortality during the acute phase of HUS was 8.6% and 24% of survivors developed end stage renal disease (ESRD) at first episode [17]. In the Italian cohort, mortality at first episode was 8,4%, ranging from 12% in children to 2% in adults, and 32% of survivors did not recover renal function at first episode [18]. Relapses of HUS are mostly observed in patients with mutations of *MCP *(70%-90% of patients have relapses) [17,18], *CFH *(50%) [17,18], *C3 *(50%) [18,83,84] and anti-CFH antibodies (40-60%) [18,37]. In MCP-mutated patients, relapses occur at unpredictable intervals of a few months to several years, most often triggered by infections. Relapses with complete recovery are characteristic of MCP-HUS in children [17,18] (Table 3).

aHUS patients have been noticed to have cardiovascular ischemic events complicating flares of the disease [18,41]. Stenoses of intra-and extracranial arteries, large thoracic and abdominal aorta branches, pulmonary and coronary arteries have been demonstrated in a 10 year-old child with aHUS and a CFB mutation [177] and cerebral artery stenoses in a 15 year-old child with CFH mutation [178]. These observations suggest that aHUS with complement dysregulation may involve large arteries and support the logic of complement inhibition by anti-C5 antibody.

## Prognosis

The data presented here come from the two main cohorts of clinically documented patients: the French pediatric cohort [17] and the Italian cohort concerning children and adults [18], which included both retrospective and recent patients, but none treated by eculizumab. The overall mid-term prognosis of aHUS was poor, and more severe in adults than in children. At 3 to 5 years after onset, 44% [17] to 48% [18] of children and 67% of adults [18] had either died or reached ESRF.

Prognosis varies according to genotype (Table 3). The worst prognosis is in patients with *CFH *mutation and the best in patients with MCP mutation. In CFH-mutated patients, mortality at first episode was 20-30% in children (a percentage now historical) and 4% in adults and evolution to ESRF at first episode in survivors 20-40% in children and 48% in adults [17,18]. By comparison, no patient with *MCP *mutation from either cohort died at first episode, none of the MCP-children and only 25% of MCP-adults developed ESRF at first episode. Among patients with *CFI *mutations, 50-60% went to ESRF at first episode or within the year after onset, while the other half have preserved renal function generally without further relapses [17,18]. At three to five years follow-up, the percentage of patients who had died or reached ESRF was approximately 75% in CFH-mutated patients and 50-60%% in CFI-mutated patients, whether adults or children [17,18]. While 38% of MCP-mutated children in the French pediatric cohort had reached ESRF at 5 years follow-up after a number of relapses, only 6% of MCP- mutated patients in the Italian registry had developed ESRF at that stage, varying from 0% in children to 25% in adults [18]. The prognosis of HUS with *C3 *[18,83] or *CFB *[18,80,81] mutation is as poor as that of CFH-HUS, whatever the age at onset. Patients with *THBD *mutations also have a poor outcome, with evolution to ESRD in 46% of patients at 1 year and 54% at 3 years follow-up [18]. Among patients with anti-CFH antibodies, 35% [37] to 60% [18] developed ESRD within 3 years follow-up.

Recent progress in diagnosis (e.g. early detection of anti-CFH antibodies) and therapeutic options, including early aggressive and prolonged plasma therapy and the use of eculizumab, most probably will allow a much better outcome of the disease. It is not unrealistic to consider that the poor prognosis indicated by presently available cohorts will soon appear as outdated.

## Conclusion

The progress in the understanding of the physiopathology of aHUS during the last decade has opened the way to new therapies which hopefully will prevent the evolution to ESRF in the patients at risk, and allow a successful transplantation in the patients presently on dialysis. Recent trials and clinical experience confirm the efficiency of the complement blocker eculizumab. The challenge is now to define the best choice for each individual patient, according to the identified complement anomaly(ies) and the phase of the disease, between plasma therapy, eculizumab, liver or combined live-kidney transplantation and, in the near future, CFH concentrate or recombinant CFH. Thanks to the progress in knowledge and comprehension, the disease has entered a new era.

## Abbreviations

ADAMTS 13: A Disintegrin And Metalloprotease with ThromboSpondin type 1 repeats 13; aHUS: atypical hemolytic uremic syndrome; APL: antiphospholipid; CFH: complement factor H; CFI: complement factor I; CFB: complement factor B; CNS: central nervous system; D+ HUS: post-diarrheal hemolytic uremic syndrome; (D-) HUS: non-post-diarrheal hemolytic uremic syndrome; Elisa, enzyme linked immunosorbent assay; ESRF: end-stage renal failure; FFP: fresh frozen plasma; HELLP syndrome: Hemolytic anemia, elevated Liver enzymes, and Low Platelets syndrome; HLA: human leukocyte antigen; HUS: hemolytic uremic syndrome; LDH: lactate deshydrogenase; MAC: membrane attack complex; MCP: membrane cofactor protein; MLPA: Multiplex Ligation dependent Probe Amplification; MRI: magnetic resonance imaging; PCR: polymerase chain reaction; PI: plasma infusion; PE: plasma exchange; RBC: red blood cell; RCA: regulators of complement activation; SCR: short consensus repeat; SLE: systemic lupus erythematosus; SNP: Single Nucleotide Polymorphism; Stx: Shiga-like toxin; STEC: Shiga-toxin producing *Escherichia coli*; *S pneumoniae*: *Streptococcus pneumoniae*; THBD: thrombomodulin; TMA: thrombotic microangiopathy; TTP: thrombotic thrombocytopenic purpura; USA: United States of America; VEGF: vascular endothelial growth factor

## Competing interests

C. Loirat has been coordinator for France of the Alexion trials "Safety and efficacy of Eculizumab in atypical HUS adult patients resistant to plasma therapy/sensitive to plasmatherapy" C08-002A and C08-003A, is coordinator for France of the trials "Eculizumab in adult patients/pediatric patients with atypical HUS" C10-004 and C10-003, and is on scientific advisory boards of Alexion Pharmaceuticals and LFB Biotechnologies. V. Frémeaux-Bacchi is on scientific advisory board of Alexion Pharmaceuticals.

## Authors' contributions

CL and VFB discussed the article's content, wrote the manuscript, reviewed it before submission and approved the final manuscript. VFB carried out complement investigations and genetic screening.
